# The Partitioning of Newly Assimilated Linoleic and α-Linolenic Acids Between Synthesis of Longer-Chain Polyunsaturated Fatty Acids and Hydroxyoctadecaenoic Acids Is a Putative Branch Point in T-Cell Essential Fatty Acid Metabolism

**DOI:** 10.3389/fimmu.2021.740749

**Published:** 2021-10-05

**Authors:** Johanna von Gerichten, Annette L. West, Nicola A. Irvine, Elizabeth A. Miles, Philip C. Calder, Karen A. Lillycrop, Barbara A. Fielding, Graham C. Burdge

**Affiliations:** ^1^ Department of Nutritional Sciences, Faculty of Health and Medical Sciences, University of Surrey, Surrey, United Kingdom; ^2^ School of Human Development and Health, Faculty of Medicine, University of Southampton, Southampton, United Kingdom; ^3^ National Institute of Health Research (NIHR) Southampton Biomedical Research Centre, University Hospital Southampton National Health Service (NHS) Foundation Trust and University of Southampton, Southampton, United Kingdom; ^4^ Centre for Biological Sciences, Faculty of Natural and Environmental Sciences, University of Southampton, Southampton, United Kingdom

**Keywords:** T lymphocyte, oxylipin, omega-3 and omega-6 polyunsaturated fatty acids, desaturase, elongase, stable isotope, FADS2, ELOVL5

## Abstract

Longer-chain polyunsaturated fatty acids (LCPUFAs) ≥20 carbons long are required for leukocyte function. These can be obtained from the diet, but there is some evidence that leukocytes can convert essential fatty acids (EFAs) into LCPUFAs. We used stable isotope tracers to investigate LCPUFA biosynthesis and the effect of different EFA substrate ratios in human T lymphocytes. CD3^+^ T cells were incubated for up to 48 h with or without concanavalin A in media containing a 18:2n-6:18:3n-3 (EFA) ratio of either 5:1 or 8:1 and [^13^C]18:3n-3 plus [d_5_]18:2n-6. Mitogen stimulation increased the amounts of 16:1n-7, 18:1n-9, 18:2n-6, 20:3n-6, 20:4n-6, 18:3n-3, and 20:5n-3 in T cells. Expression of the activation marker CD69 preceded increased *FADS2* and *FADS1* mRNA expression and increased amounts of [d_5_]20:2n-6 and [^13^C]20:3n-3 at 48 h. In addition, 22-carbon n-6 or n-3 LCPUFA synthesis was not detected, consistent with the absence of *ELOVL2* expression. An EFA ratio of 8:1 reduced 18:3n-3 conversion and enhanced 20:2n-6 synthesis compared to a 5:1 ratio. Here, [d_5_]9- and [d_5_]-13-hydroxyoctadecadienoic (HODE) and [^13^C]9- and [^13^C]13-hydroxyoctadecatrienoic acids (HOTrE) were the major labelled oxylipins in culture supernatants; labelled oxylipins ≥20 carbons were not detected. An EFA ratio of 8:1 suppressed 9- and 13-HOTrE synthesis, but there was no significant effect on 9- and 13-HODE synthesis. These findings suggest that partitioning of newly assimilated EFA between LCPUFA synthesis and hydroxyoctadecaenoic acid may be a metabolic branch point in T-cell EFA metabolism that has implications for understanding the effects of dietary fats on T lymphocyte function.

## Introduction

Leukocyte membranes are characterised by high proportions of polyunsaturated fatty acids (PUFAs), in particular, arachidonic acid (20:4n-6), which are important regulators of immune cell function. Such regulation is mediated by the biophysical properties of cell membranes acting *via* the activities of membrane-associated proteins (1) and synthesis of lipid second messengers including eicosanoids derived from 20:4n-6 for example 2-series prostaglandins (PG) and 4-series leukotrienes (2–8), biologically less active 3-series PG from eicosapentaenoic acid (20:5n-3) (9–11) and oxylipins derived from 18:2n-6 or 18:3n-3, namely hydroxyoctadecadienoic (HODE) and hydroxyoctadecatrienoic acids (HOTrE) (12), and dihydroxyoctadecaenoic (DiHOME) and dihydroxyoctadecadienoic acid (DiHODE) (13). Diacylglycerol and phosphatidic acid with different PUFA compositions can differentially activate specific protein kinase C isoforms (14). Moreover, 20:5n-3, docosapentaenoic acid (22:5n-3), and docosahexaenoic acid (22:6n-3) are substrates for the synthesis of specialised pro-resolving mediators, namely, resolvins, protectins, and maresins *via* the actions of cyclooxygenase and lipoxygenases (15, 16). PUFA can modify transcription *via* the activities of ligand-activated transcription factors, primarily those of the peroxisome proliferator-activated receptor family (17), and by inducing changes in epigenetic processes (18). Therefore, induction and resolution of the immune response requires an adequate and timely supply of PUFA.

Leukocyte activation involves differential changes in the PUFA content of cell membranes (19–21). For example, mitogen activation of human T cells increased the proportions of oleic acid (18:1n-9), 22:5n-3, and 22:6n-3 and decreased the proportions of 20:1n-9 and 20:2n-6 that together were associated with altered membrane fluidity (22). These changes involve increased activities of phospholipid acyl-remodelling processes (23–25).

Since an appropriate membrane fatty acid composition is important for normal T-cell function, it is important to understand how requirements for specific fatty acids are met. Leukocytes can obtain preformed PUFA from their environment by a mechanism that is upregulated in activated cells but does not appear to exhibit a preference for PUFA (24). Dietary supplementation can induce a dose-related increase in the proportions of 20:5n-3 and 22:6n-6 in peripheral blood mononuclear cells (PBMCs) (26, 27). Such changes in cell membrane composition can be associated with changes in immune function (28) that are due, at least in part, to exchange of 20:4n-6 with 20:5n-3 in cell membranes and altered patterns of production of more and less pro-inflammatory lipid mediators (2–4).

The pathway for synthesis of longer-chain PUFAs from essential fatty acids (EFAs) was first demonstrated in rat hepatocytes involving a series of desaturation and elongation reactions and a final single cycle of peroxisomal fatty acid *β*-oxidation (29, 30). The initial rate-limiting reaction is catalysed by Δ6 desaturase, encoded by *FADS2*, which preferentially desaturates 18:3n-3 compared to 18:2n-6, followed by chain elongation by elongase-5, encoded by *ELOVL5*, and Δ5 desaturation by Δ5 desaturase, which is encoded by *FADS1*. The carbon chain then undergoes two rounds of elongation catalysed by elongase-2 and/or elongase 5. This is followed by further desaturation by Δ6 desaturase, translocation of the 24 carbon intermediates to peroxisomes, and shortening by one cycle of *β*-oxidation (29).

PUFA synthesis in activated leukocytes proceeds by a modified pathway, depending on cell type, compared to that reported in hepatocytes. Mitogen stimulation of PBMCs or T lymphocytes was associated with induction of Δ9, Δ6, and Δ5 desaturase activities, although the synthesis of PUFA was not characterised directly (31). However, it was concluded that these increased enzyme activities were insufficient to explain the changes in membrane composition in activated leukocytes (31). Others have shown that activated murine macrophages are unable to convert 18:2n-6 to 20:4n-6 and that these cells lack Δ6 desaturase activity, although elongation of 18:2n-6 to 20:2n-6 was detected (32). Furthermore, murine macrophages elongated 18:3n-3 to 20:3n-6, which was a substrate for synthesis of PGE_1_ (32). Such capacity for PUFA biosynthesis may be one means of ensuring timely supply of PUFA substrates for synthesis of lipid mediators (33). However, it is not known whether there is preferential use of newly synthesised LCPUFA for the synthesis of lipid mediations compared to the bulk fatty acid pools.

Mitogen stimulation of human PBMCs induced upregulation of FADS 1 and 2, and ELOVL5 and ELOVL4 mRNA expression, together with increased uptake of [^13^C]18:3n-3 and conversion to 20:3n-3, 20:4n-3, 20:5n-3, and 22:5n-3 in a sex-independent manner (34). Synthesis of 18:4n-3 and 22:6n-3 was not detected. Thus, the initial reactions were reversed compared to the pathway described previously (29) in that the first reaction was elongation, which has been suggested to involve elongase-5 activity (35), followed by Δ8 desaturation possibly catalysed by the protein product of mammalian *FADS2*, which has been shown to catalyse both Δ6 and Δ8 desaturation when transfected into yeast (36) and in Jurkat T lymphocyte leukaemia cells incubated with [^13^C]18:3n-3 (34). Others have reported an increase in putative Δ6 desaturation products in human activated T lymphocytes incubated with either 18:2n-6 or 18:3n-3 (35), although because these findings were not based on fatty acid tracers, they do not exclude the possibility of selective uptake and utilisation of preformed LCPUFA from the medium instead of conversion of EFA. Thus, there is uncertainty about the nature of PUFA biosynthesis during T-cell activation and its contribution to the changes in cellular fatty acid composition.

The first two reactions of the hepatic PUFA synthesis pathway are reversed in PBMCs and T cells (34, 35), such that the first step is carbon chain elongation followed by Δ8 desaturation into 20:3n-3 and 20:2n-6. One possible interpretation is the rate-limiting reaction, and selectivity for 18:3n-3 and 18:2n-6 differs between leukocytes and hepatocytes. To address this, [d_5_]18:2n-6 and [^13^C]18:3n-3 tracers were used to characterise PUFA biosynthesis in human CD3^+^ T lymphocytes during the first 48 h after mitogen activation in the presence of differing EFA ratios of 18:2n-6 to 18:3n-3 that are representative of relative EFA intakes in western populations (37).

## Materials and Methods

### Ethics Statement

The study was reviewed and approved by the East of England-Cambridge Central Research Ethics Committee (approval number 19/EE/0096), and all participants gave written informed consent.

### Participants and Collection of Blood Samples

Inclusion criteria for the study were 18–30 years, body mass index 18.5 and 30.0 kg/m^2^, systolic blood pressure ≤140 mm/Hg, diastolic blood pressure ≤90 mm/Hg, random total cholesterol concentration <7.5 mmol/L, HbA1c concentration <42 mmol/mol (or <6%), C-reactive protein (CRP) concentration <3 mg/L, not consuming fish oil or other oil or dietary supplements, non-smoking, absence of chronic disease, willingness to adhere to the study protocol, and being able to provide written informed consent. Volunteers were excluded from the study if they did not meet the inclusion criteria, were pregnant or planning to become pregnant within the study period, or were participating in another clinical trial. Non-fasting venous blood samples (100 ml) were collected into vials containing lithium heparin anticoagulant on three occasions separated by an interval of 4 weeks. Participants were 10 healthy women aged 26.2 ± 0.8 years with body mass index 23.1 ± 0.4 kg/m^2^ and blood pressure (systolic 106 ± 3 mmHg; diastolic 62.7 ± 2 mmHg), total plasma cholesterol (4.0 ± 0.2 mmol/l), CRP (1.1 ± 0.1 mg/L), and HbA1c (30.5 ± 1.0 mmol/L) concentrations within normal ranges.

### Isolation and Culture of CD3^+^ T Cells From Whole Blood

Whole blood was layered onto a histopaque density cushion, and erythrocytes and granulocytes were removed by centrifugation at 845 × g for 15 min at room temperature. PBMCs were collected by aspiration and diluted 1:1 with RPMI1640 containing 10% (v/v) autologous pooled heat-inactivated serum. CD3^+^ T cells were isolated by negative selection using the T-cell EasySep kit (StemCell Technologies) as instructed by the manufacturer. Isolated T cells were washed with 10 ml of RPMI1640 containing 10% (v/v) autologous pooled heat-inactivated serum and collected by centrifugation at 300 × g for 10 min at room temperature. Cryopreservation was carried out as described (38, 39). Ice-cold RPMI1640 medium containing 20% (v/v) dimethylsulphoxide and 10% fetal bovine serum was added to the T-cell pellet, and the cells were frozen at -80°C overnight and then transferred to liquid nitrogen for storage until used.

T-cell culture was carried out essentially as described (34). Briefly, cryopreserved cells were thawed and resuspended in RPMI1640 containing 2 mM of L-glutamine, 100 units/ml of penicillin, and 100 µg/ml of streptomycin and 10% (v/v) heat-inactivated pooled human serum (Sigma-Aldrich) (**Supplementary Table S1**) and adjusted to a density of 1 × 10^6^ cells/ml. The fatty acid composition of the medium was standardised by using pooled homologous serum instead of autologous serum. The fatty acid composition of the medium was adjusted by addition of 18:2n-6 or 18:3n-3 as free fatty acids to a final ratio of either 8:1 or 5:1 (EFA ratio; **Table 1**). Total 18:2n-6 and 18:3n-3 concentrations included ethyl-[d_5_]18:2n-6 (2 µmol/L) and [1-^13^C]18:3n-3 (2 or 4 µmol/L, according to the EFA ratio 8:1 or 5:1, respectively). In order to test whether 18:2n-6 metabolism was altered by the presence of an esterified ethyl group, uptake and conversion of ethyl-[d_5_]18:2n-6 (2 µmol/L) were compared to those of unesterified [d_5_]18:2n-6 (2 µmol/L) in cultures with an EFA ratio of 5:1 (n = 5 per molecular form with or without mitogen stimulation). Cultures were placed in a humidified incubator in an atmosphere containing 5% (v/v) CO_2_ for up to 48 h with or without concanavalin A (Con A; 10 μg/ml; Sigma-Aldrich). Cells were collected by centrifugation and washed as before, and then either snap-frozen and stored at −80°C or processed immediately by flow cytometry.

**Table 1:** Cell culture medium fatty acid composition.

EFA ratio	Concentration (µmol/L)	
	5:1	8:1
14:0	8.2	7.7
16:0	148.0	169.0
18:0	47.0	61.5
20:0	0.3	0.2
16:1n-7	11.0	13.3
18:1n-9	113.1	131.8
18:1n-7	8.2	9.7
20:1n-9	1.2	0.8
18:2n-6	162.5	209.2
18:3n-6	1.5	2.1
20:2n-6	1.0	1.2
20:3n-6	6.2	8.3
20:4n-6	22.0	29.9
18:3n-3	35.8	27.3
20:3n-3	0.3	0.4
20:4n-3	1.0	2.1
20:5n-3	1.1	1.6
22:5n-3	1.6	1.6
22:6n-3	2.7	3.0
Total SFA	203.5	238.4
Total MUFA	133.5	155.7
Total n-6 PUFA	30.8	41.5
Total n-3 PUFA	6.6	8.7

Total SFA, sum of all saturated fatty acids; total MUFA, sum of all monounsaturated fatty acids; total n-6 PUFA, sum of all n-6 polyunsaturated fatty acids excluding 18:2n-6; total n-3 PUFA, sum of all n-3 polyunsaturated fatty acids excluding 18:3n-3; EFA, essential fatty acid. The 18:2n-6-to-18:3n-3 ratio was adjusted to 5:1 and 8:1 by addition of free EFA.

T-cell activation was assessed by the cell surface expression of CD69 as described (34, 40). Briefly, cells were incubated with PE-Cy7-conjugated anti-human CD69 monoclonal antibody (catalogue number 557745, BD Biosciences) for 30 min at 4°C in the dark, processed for flow cytometry, and analysed using a FACSCalibur (B&D Biosciences) flow cytometer as described (34).

### Analysis of Media and T-Cell Fatty Acid Composition by Gas Chromatography

Culture medium (0.9 ml) was thawed, and purified T cells were thawed and suspended in 0.9% (v/v) NaCl. Then, 17:0 (3 µg) was added as internal standard to purified T cells, and 10 µg of 17:0 internal standard was added to culture medium samples. Total lipids were extracted with chloroform/methanol (2:1, v/v) (41). Fatty acid methyl esters (FAMEs) were synthesised from total cell and culture medium lipids by incubation with methanol containing 2% (v/v) sulphuric acid at 50°C for 2 h (42). The reaction mixture was cooled to room temperature and neutralised with a solution of KHCO_3_ (0.25 M) and K_2_CO_3_ (0.5 M). FAMEs were collected by extraction with hexane (42).

FAMEs were resolved on a BPX-70 fused silica capillary column (30 m × 0.25 mm × 25 μm) fitted in an Agilent 6890 gas chromatograph equipped with flame ionisation detection (GC-FID) (43). FAMEs (1 μl) were injected in split mode at an inlet temperature of 300°C with helium carrier at a flow rate of 1 ml/min (44). The oven temperature was held at 115°C for 2 min post-injection, then increased at 10°C/min to 200°C and held for 16 min. The oven temperature was then increased at 60°C/min to 240°C and held for 2 min. The detector was maintained at 300°C. Chromatograms were integrated manually using ChemStation software (version B.03.01, Agilent Technologies), and the amount of cellular fatty acids, expressed as nmol/10^6^ cells, was calculated by comparison of the peak area of each fatty acid of interest to that of the internal standard and adjusted for the number of cells that were extracted. Fatty acid concentrations in culture media were calculated by comparison of the peak area of each fatty acid of interest to that of the internal standard and adjusted for the volume of media that was extracted. Fatty acids were identified by their retention times relative to standards (37 FAMEs, Sigma-Aldrich).

### Analysis of T-Cell Fatty Acid Composition and Stable Isotope Enrichment by Gas Chromatography–Mass Spectrometry

The purity of FAMEs from T-cell extractions was tested by gas chromatography (GC)–mass spectrometry using a mass scan m/z 50–550. Samples were reconstituted in 50 µl of hexane, and a 1-µl injection volume in splitless mode was used with a column flow of 1.5 ml/min. FAMEs were resolved on a Supelcowax 10 capillary column (30 m × 0.25 mm × 0.25 μm film thickness; Supelco) on a 6890 gas chromatograph (Agilent, UK) equipped with a mass selective detector (Agilent 5975). The inlet and detector were set to 250°C, and helium was used as carrier gas. The temperature gradient was set to start at 60°C and held for 3 min, then raised at 12°C/min to 200°C and held for 6 min, followed by a second increase at 12°C/min to 240°C and finally held for 15 min. Fatty acids were compared by their retention times relative to authentic standards (FAME37 Restek 35077) and mass spectra confirmed with the National Institute of Standards and Technology database (45). The presence and synthesis of 18:3n-6 and 18:4n-6 were examined by comparison of the fragmentation spectra within the predicted retention time window of authentic fatty acid standards.

### Analysis of Stable Isotope Enrichment of Fatty Acids by Gas Chromatography–Isotope Ratio Mass Spectrometry

In this study, [d_5_] or [1-^13^C] enrichment of n-6 or n-3 PUFA, respectively, was measured by GC-thermal conversion or combustion–isotope ratio mass spectrometry as described (46). Briefly, FAMEs were reconstituted in 25 µl of hexane and a 2-µl injection volume in splitless mode onto a Supelcowax 10 capillary column (30 m length × 0.25 mm diameter × 0.25 μm film thickness; Supelco) run with a column flow of 1.5 ml/min on a Thermo Trace 1310 gas chromatograph (ThermoFisher, Loughborough, UK) equipped with a high-temperature (1,000°C/1,400°C) combustion/thermal conversion furnace and a Thermo Delta V IRMS. The ^13^C/^12^C and ^2^H/^1^H ratios for identified fatty acids were measured relative to laboratory reference gas standards calibrated to the international standards (Vienna Standard Mean Ocean Water or Vienna Pee Dee Belemnite, respectively). Stable isotope enrichment was calculated as described (47). The concentration of each labelled fatty acid was calculated from the amount measured by GC-FID normalised to 1 million cells.

### Analysis of Stable Isotope Enrichment of Oxylipins by Liquid Chromatography–Mass Spectrometry

The supernatant from unstimulated or mitogen-stimulated T cells cultured for 48 h in medium containing an EFA ratio of 8:1 was collected and immediately frozen at -80°C. Free oxylipins were isolated by solid-phase extraction (SPE) (48). Briefly, supernatants (1–2 ml) were defrosted at 4°C overnight. An antioxidant mix containing butylated hydroxytoluene and EDTA (both 0.2 mg/ml), indomethacin (100 µM), and 4-[[trans-4-[[(tricyclo[3.3.1.13,7]dec-1-ylamino)carbonyl]amino]cyclohexyl]oxy]-benzoic acid (100 µM) in methanol/water 1:1 (v/v) (40 µl) was added to the frozen supernatant (49). The internal standard [d_5_](17(*S*)-hydroxydocosa-4,7,10,13,15,19-hexaenoic-21,21,22,22,22-d5-acid) ([d_5_]17-HDHA; 20 ng) was added, and proteins were then precipitated with 750 µl of ice-cold methanol for 30 min at -20°C. Samples were acidified with 1 M HCl (10 µl) and applied to Oasis HLB (Waters) SPE cartridges, washed with 10% (v/v) methanol in water, pure water, and hexane, and oxylipins were then each eluted with 100% ethyl acetate and methanol. Eluates were pooled, dried under nitrogen, and stored in 100 µl of methanol/water 70:30 (v/v) at -20°C before liquid chromatography–mass spectrometry (LCMS) analysis within 48 h. Oxylipins were analysed with multiple reaction monitoring (MRM) using an Acquity I-class and Xevo TQS UPLC-MS/MS system (Waters). Negative electrospray ionisation parameters were as follows: 2.4 kV capillary voltage, 40 V cone voltage, 600°C desolvation temperature, 1,000 L/h desolvation flow, 150 L/h cone flow, and 7 bar nebuliser pressure. MRM transitions are shown in **Supplementary Table S1**.

Lipids were separated using a Cortecs C18 (2.1 mm × 100 mm, 1.6 µm) column (Waters) with a BEH C18 VanGuard (2.1 mm × 5 mm, 1.7 µm) pre-column (Waters) at 40°C with the autosampler temperature at 10°C and a flow rate of 0.3 ml/min. The mobile phase A was 80:20 (v/v) water/acetonitrile and mobile phase B 75:25 (v/v) acetonitrile/methanol, both containing 0.02% (v/v) formic acid. The linear gradient started with 20% mobile phase B for 1 min, increased to 35% B for 2 min, and further increased to 70% B for 7 min, with a final increase to 95% B for 2 min and a hold time of 2 min until decreasing back to 20% B for 2 min with an additional 2-min conditioning phase.

The limits of detection were 9.9 pg/µl of [d_5_]17-HDHA and 9.0 pg/µl of [d_4_]9-hydroxy-10(*E*),12(*Z*)-octadecadienoic acid (9(*S*)HODE) (**Supplementary Figure S1**). SPE recovery and quality control coefficient of variation of [d_4_]9(*S*)-HODE were 67% ± 10% and ± 18.1%, respectively (**Supplementary Figure S1**). Data were processed using MassHunter 4.0 (Waters). Oxylipin concentrations were calculated relative to the internal standard [d_5_]17-HDHA, then normalised to supernatant volume and background-corrected with data from the 48-h cell supernatant absent of T cells, and then normalised to the number of T cells in the cell culture.

### Analysis of mRNA Expression by Real-Time RTPCR

mRNA expression of genes that encode enzymes involved in the PUFA synthesis pathway was carried out as described. Briefly, total T-cell RNA was extracted using the Qiagen RNeasy Mini kit (Qiagen) combined with on-column DNase digestion (Qiagen) as instructed by the manufacturer. RNA was eluted in RNase-free water (30 μl). RNA concentration was measured, and purity was assessed using a NanoDrop1000 spectrophotometer. RNA integrity was confirmed by agarose gel electrophoresis. cDNA was synthesised by reverse transcription, and real-time RTPCR was carried out using primers listed in **Supplementary Table S2**. Amplified transcripts were quantified using the standard curve method (50) and normalised to the geometric mean of the reference genes 60S ribosomal protein L13-A (*RPL13A*) and succinate dehydrogenase complex, subunit A, flavoprotein variant (*SDHA*), which were shown to be stable across culture conditions by the GeNorm method (51). *ELOVL2* and *ELOVL4* mRNA expression were assessed by agarose gel electrophoresis. Briefly, the respective transcripts were amplified by 40 PCR cycles. RNA from HepG2 cells or Jurkat cells was used as a reference for *ELOVL2* and *ELOVL4*, respectively. PCR products were resolved on 2% (w/v) agarose gel containing GelRed and visualised under UV light.

### Statistical Methods

Data were analysed by one-way or two-way ANOVA with single-factor effects of time after stimulation and EFA ratio and with two-factor interaction effects using SPSS version 27 (IBM SPSS Statistics for Windows, Version 27.0; IBM Corp., Armonk, NY, USA). In some experiments, cell activation was included as an additional fixed factor. *Post-hoc* pairwise comparisons were done by Tukey’s test. Statistical significance was assumed at p < 0.05. The magnitude of the effect size (ηp^2^) was ≥0.14 for all statistically significant single-factor and interaction effects. Comparisons between unstimulated and stimulated cultures within each time point and EFA ratio were by Student’s paired t-test adjusted for multiple comparisons by the Holm–Sidak method using GraphPad Prism (Version 8 for Windows, GraphPad Software, San Diego, CA, USA; www.graphpad.com).

## Results

### Effect of Mitogen Stimulation on the Cell Surface Expression of CD69

**Figure 1:**
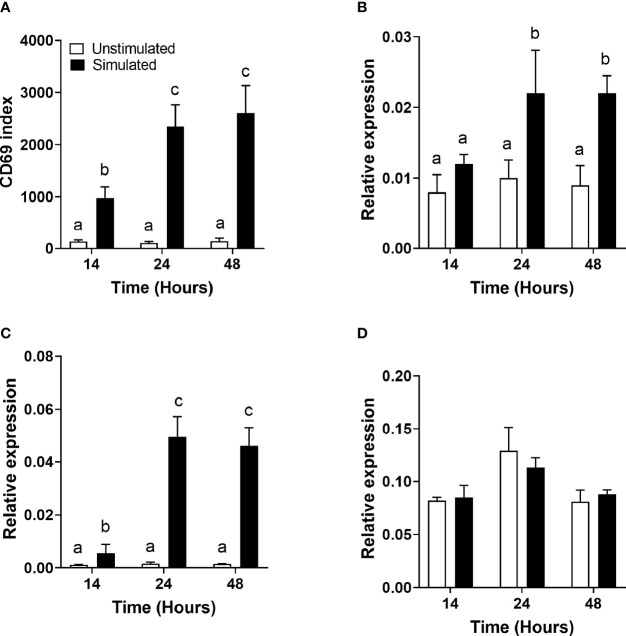
The effect of duration of incubation and mitogen stimulation on the cell surface expression of CD69 and the mRNA expression of genes involved in polyunsaturated fatty acid biosynthesis in T cells. Values are mean ± SEM (n = 10/time point) **(A)** CD69 index (proportion of cells in the positive gate multiplied by the median fluorescence intensity) after 14-, 24-, and 48-h incubation; **(B)** relative expression of *FADS1* mRNA; **(C)** relative expression of *FADS2* mRNA; and **(D)** relative expression of *ELOVL5* mRNA. Means represented by bars with different letters were significantly different by two-way ANOVA with Tukey’s *post-hoc* test.

There was a significant single-factor effect of time [F(2,53) = 10.44, p = 0.04] and stimulation [F(2,53) = 63.23, p < 0.0001], but no significant time * stimulation interaction (p = 0.63), on CD69 expression (**Figure 1A**). The CD69 index (the ratio of the mean fluorescence intensity to the number of events in the positive region) was significantly greater in stimulated than unstimulated cells at all time points measured and increased significantly between 14 and 24 h.

### Effect of Mitogen Stimulation on the mRNA Expression of Desaturases and Elongases Involved in Polyunsaturated Fatty Acid Biosynthesis

**Figure 2:**
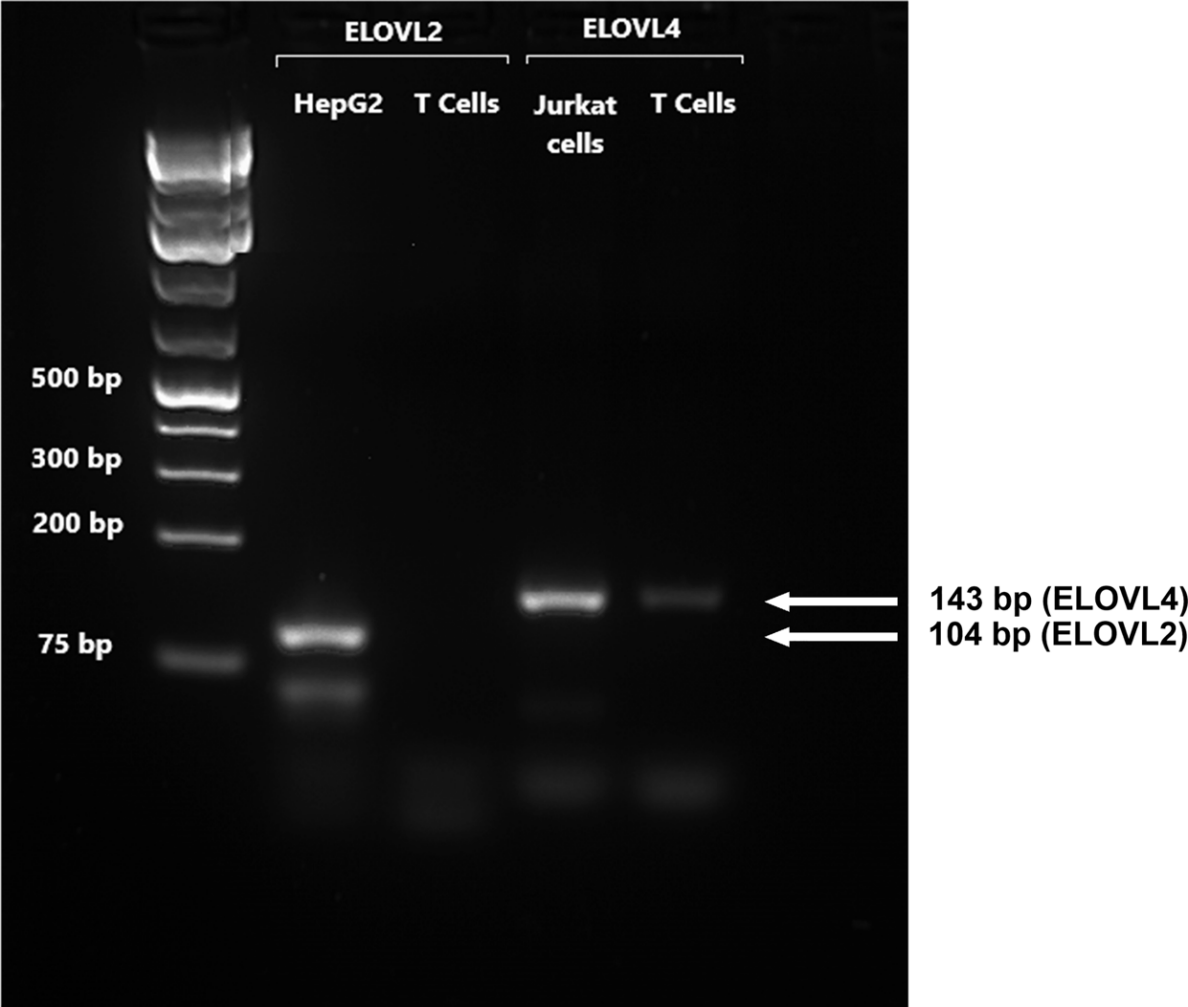
A representative RTPCR analysis of the mRNA expression of *ELOVL2* and *ELOVL4* in stimulated T cells after 48-h incubation. Bands correspond to PCR products after 40 cycles using T-cell, HepG2, and Jurkat-cell cDNA as templates. HepG2 cells were used as reference for *ELOVL2*, as this gene is poorly expressed in Jurkat cells. Jurkat cells were used as the reference for *ELOVL4*, which is poorly expressed in liver cells.

*FADS1* mRNA expression increased between 14 and 24 h and was significantly greater in stimulated than unstimulated cells at 24 and 48 h (**Figure 1B**). There were significant single-factor effects of time [F(2,54) = 2.17, p = 0.01] and cell stimulation [F(2,54) = 13.69, p = 0.001] and a significant time * stimulation interaction [F(2,54) = 1.2, p = 0.03] on *FADS1* mRNA expression. *FADS2* expression was significantly greater in stimulated than unstimulated cells at 24 and 48 h and increased significantly between 14 and 24 h. There were significant single-factor effects of time [F(2,54) = 12.41, p < 0.0001] and cell stimulation [F(2.54) = 50.38, p < 0.0001] and a significant time * cell stimulation interaction effect [F(2,54) = 11.8, p < 0.0001] on *FADS2* mRNA expression (**Figure 1C**). There were no significant single-factor effects of time or cell stimulation on *ELOVL5* mRNA expression (**Figure 1D**). *ELOVL2* expression was below the level of detection by real-time RTPCR and agarose gel electrophoresis (**Figure 2**). *ELOVL4* expression was also below the level of detection by real-time RTPCR, although a faint band of molecular weight that corresponded to the PCR product of *ELOVL4* was detected by agarose gel electrophoresis (**Figure 2**).

### Effect of Mitogen Activation, EFA Ratio, and Duration of Culture on T Lymphocyte Fatty Acid Composition

Stimulation with Con A increased the amounts of the monounsaturated fatty acids 16:1n-7, 18:1n-9, and 18:1n-7 compared to unstimulated cells cultured in the presence of EFA ratios of 5:1 or 8:1 but did not alter the amounts of saturated fatty acids or 20:1n-9 (**Table 2**). The amount of 18:2n-6 was significantly greater (approximately 2-fold) in stimulated cells than unstimulated cells at 48 h regardless of the EFA content of the medium. The amounts of 20:3n-6 and 20:4n-6 were significantly greater after 48 h (approximately 1.2-fold and 1.1-fold, respectively) regardless of the EFA ratio in the culture medium (**Table 2**). There was no significant effect of mitogen stimulation on the amount of 20:2n-6 or 22:4n-6.

**Table 2:** The effect of mitogen stimulation on T-cell total fatty acid composition.

	14 h			24 h			48 h		
	nmol/10^6^ cells		t-test	nmol/10^6^ cells		t-test	nmol/10^6^ cells		t-test
	U	S	adjP	U	S	adjP	U	S	adjP
	EFA ratio (5:1)								
14:0	0.95 ± 0.17	0.87 ± 0.17	>0.999	0.51 ± 0.15	0.47 ± 0.09	0.818	0.96 ± 0.17	0.92 ± 0.21	0.891
16:0	3.17 ± 0.24	4.31 ± 0.29	>0.999	3.70 ± 0.40	4.22 ± 0.40	0.372	3.27 ± 0.23	4.79 ± 0.64	0.460
18:0	2.90 ± 0.03	3.12 ± 0.32	>0.999	3.15 ± 0.33	3.10 ± 0.34	0.901	2.40 ± 0.10	3.02 ± 0.44	0.206
20:0	0.06 ± 0.00	0.06 ± 0.01	>0.999	0.03 ± 0.00	0.03 ± 0.01	0.991	0.02 ± 0.00	0.02 ± 0.00	0.427
16:1n-7	0.26 ± 0.31	0.18 ± 0.01	>0.999	0.14 ± 0.02	0.17 ± 0.02	0.255	0.12 ± 0.01	0.23 ± 0.03	0.005
18:1n-9	1.97 ± 0.33	2.54 ± 0.20	>0.999	2.31 ± 0.32	2.58 ± 0.27	0.525	1.68 ± 0.14	3.08 ± 0.39	0.007
18:1n-7	0.09 ± 0.01	0.28 ± 0.02	>0.999	0.24 ± 0.02	0.29 ± 0.02	0.152	0.19 ± 0.01	0.28 ± 0.03	0.007
20:1n-9	0.29 ± 0.03	0.06 ± 0.00	0.9999	0.05 ± 0.00	0.04 ± 0.00	0.632	0.04 ± 0.00	0.05 ± 0.00	0.152
18:2n-6	0.32 ± 0.02	0.20 ± 0.13	0.9999	1.18 ± 0.08	2.07 ± 0.13	<0.0001	1.34 ± 0.10	2.65 ± 0.18	<0.0001
18:3n-6	n.d.	n.d.		n.d.	n.d.		n.d.	n.d.	
20:2n-6	0.16 ± 0.19	0.12 ± 0.05	0.9998	0.09 ± 0.02	0.09 ± 0.02	0.983	0.06 ± 0.01	0.09 ± 0.02	0.273
20:3n-6	0.07 ± 0.01	0.27 ± 0.02	0.9999	0.18 ± 0.01	0.21 ± 0.01	0.104	0.18 ± 0.01	0.22 ± 0.01	0.035
20:4n-6	0.03 ± 0.01	0.52 ± 0.12	0.9999	1.24 ± 0.04	1.41 ± 0.05	0.013	1.25 ± 0.04	1.37 ± 0.08	0.019
22:4n-6	0.32 ± 0.24	0.13 ± 0.02	>0.999	0.14 ± 0.03	0.12 ± 0.02	0.549	0.18 ± 0.02	0.20 ± 0.04	0.576
18:3n-3	0.13 ± 0.01	0.30 ± 0.02	>0.999	0.30 ± 0.04	0.37 ± 0.06	0.351	0.25 ± 0.02	0.40 ± 0.05	0.032
18:4n-3	n.d.	n.d.		n.d.	n.d.		n.d.	n.d.	
20:3n-3	0.02 ± 0.00	0.07 ± 0.01	0.9999	0.06 ± 0.01	0.04 ± 0.01	0.075	0.04 ± 0.00	0.05 ± 0.01	0.397
20:4n-3	0.12 ± 0.01	0.02 ± 0.00	>0.999	0.03 ± 0.01	0.02 ± 0.01	0.358	0.01 ± 0.00	0.01 ± 0.00	0.559
20:5n-3	0.17 ± 0.02	0.02 ± 0.00	>0.999	0.02 ± 0.00	0.02 ± 0.00	0.330	0.02 ± 0.00	0.03 ± 0.00	0.025
22:5n-3	0.26 ± 0.31	0.16 ± 0.01	>0.999	0.11 ± 0.00	0.12 ± 0.01	0.360	0.11 ± 0.01	0.12 ± 0.01	0.874
22:6n-3	0.29 ± 0.03	0.18 ± 0.02	>0.999	0.11 ± 0.01	0.13 ± 0.02	0.402	0.12 ± 0.01	0.13 ± 0.01	0.725
	EFA ratio (8:1)								
14:0	1.28 ± 0.31	0.47 ± 0.06	0.998	0.73 ± 0.18	0.95 ± 0.20	0.253	0.47 ± 0.06	0.54 ± 0.08	0.508
16:0	3.02 ± 0.28	4.00 ± 0.32	1.000	3.97 ± 0.34	2.99 ± 0.16	0.10	4.00 ± 0.32	4.75 ± 0.26	0.085
18:0	2.23 ± 0.16	2.91 ± 0.23	0.997	2.98 ± 0.27	2.06 ± 0.09	0.143	2.91 ± 0.23	3.05 ± 0.18	0.654
20:0	0.04 ± 0.00	0.04 ± 0.01	0.997	0.10 ± 0.03	0.03 ± 0.00	0.639	0.04 ± 0.01	0.05 ± 0.01	0.205
C16:1n-7	0.11 ± 0.02	0.13 ± 0.02	1.000	0.09 ± 0.01	0.12 ± 0.01	0.001	0.13 ± 0.02	0.19 ± 0.01	0.01
C18:1n-9	1.33 ± 0.19	1.66 ± 0.18	0.998	1.55 ± 0.15	1.51 ± 0.07	0. 04	1.66 ± 0.18	2.45 ± 0.16	0.004
C18:1n-7	0.17 ± 0.01	0.19 ± 0.02	1.000	0.20 ± 0.01	0.17 ± 0.01	0.012	0.19 ± 0.02	0.25 ± 0.01	0.007
C20:1n-9	0.05 ± 0.01	0.05 ± 0.00	1.000	0.05 ± 0.01	0.05 ± 0.00	0.451	0.05 ± 0.00	0.12 ± 0.06	0.281
C18:2n-6	1.31 ± 0.20	1.69 ± 0.14	0.998	1.20 ± 0.07	1.49 ± 0.11	<0.0001	1.69 ± 0.14	2.71 ± 0.18	<0.0001
C18:3n-6	n.d.	n.d.		n.d.	n.d.		n.d.	n.d.	0.088
C20:2n-6	0.06 ± 0.01	0.16 ± 0.03	0.990	0.23 ± 0.04	0.05 ± 0.00	0.161	0.16 ± 0.03	0.23 ± 0.03	0.127
C20:3n-6	0.18 ± 0.01	0.19 ± 0.01	1.000	0.19 ± 0.01	0.17 ± 0.01	0.320	0.19 ± 0.01	0.24 ± 0.02	0.02
C20:4n-6	1.09 ± 0.07	1.31 ± 0.06	1.000	1.12 ± 0.05	1.07 ± 0.04	0.083	1.31 ± 0.06	1.43 ± 0.06	0.012
C22:4n-6	0.13 ± 0.01	0.14 ± 0.01	0.978	0.19 ± 0.03	0.13 ± 0.01	0.187	0.14 ± 0.01	0.14 ± 0.01	0.850
C18:3n-3	0.19 ± 0.02	0.19 ± 0.03	0.994	0.19 ± 0.03	0.16 ± 0.01	0.053	0.19 ± 0.03	0.29 ± 0.04	0.007
C18:4n-3	n.d.	n.d.		n.d.	n.d.		n.d.	n.d.	
C20:3n-3	0.05 ± 0.02	0.02 ± 0.00	0.997	0.02 ± 0.01	0.03 ± 0.00	0.230	0.02 ± 0.00	0.04 ± 0.01	0.113
C20:4n-3	0.00 ± 0.00	0.01 ± 0.01	>0.999	0.01 ± 0.00	0.00 ± 0.00	0.217	0.01 ± 0.01	0.01 ± 0.00	0.756
C20:5n-3	0.05 ± 0.00	0.04 ± 0.01	0.875	0.04 ± 0.01	0.04 ± 0.00	0.106	0.04 ± 0.01	0.05 ± 0.00	0.397
C22:5n-3	0.11 ± 0.01	0.11 ± 0.01	1.000	0.09 ± 0.00	0.10 ± 0.01	0.056	0.11 ± 0.01	0.13 ± 0.01	0.19
C22:6n-3	0.16 ± 0.01	0.13 ± 0.01	0.997	0.11 ± 0.01	0.15 ± 0.01	0.435	0.13 ± 0.01	0.13 ± 0.01	0.951

Values are mean ± SEM (n = 10 paired samples at each time point). Comparisons between unstimulated and stimulated cells were done by Student’s paired t-test, and statistical significance was assumed at p < 0.05. Adjustment for multiple t-tests was by the Holm–Sidak method (adjP). EFA ratio (18:2n-6:18:3n-3 in the culture medium); S, mitogen-stimulated cells; U, unstimulated cells; n.d., not detected; EFA, essential fatty acid.

**Figure 3:**
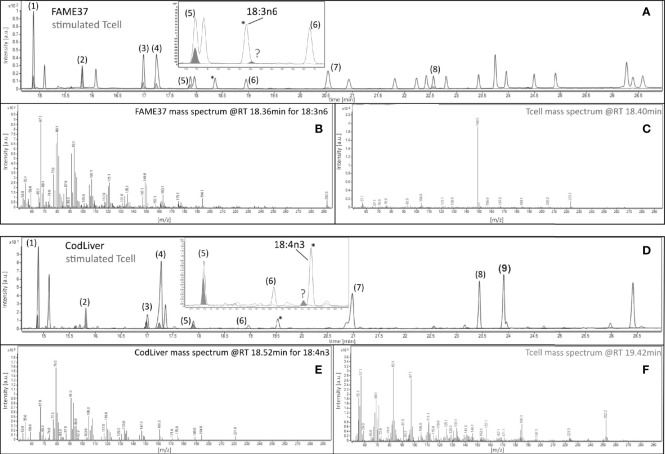
Gas chromatography–mass spectrometric analysis of 18:3n-6 and 18:4n-3 in 48-h mitogen-stimulated CD3^+^ T cells. Chromatographic separation of fatty acid methyl esters (FAMEs) from **(A)** the 37 FAMEs standard mixture and **(D)** cod liver (gray line), and from stimulated T cells (gray fill) after 48-h incubation with concanavalin A (Con A). Peaks were (1) 16:0, (2) 17:0 internal standard, (3) 18:0, (4) 18:1n-9, (5) 18:2n-6, (6) 18:3n-3, (7) 20:1n-9, (8) 20:4n-6 (FAME37), and (9) 20:5n-3 (cod liver standard). Insets show the positions of the closest peaks in T cells to authentic 18:3n-6 or 18:4n-3 peaks in 37 FAMEs standard (peaks marked by )? **(B, E)** Mass spectra of authentic 18:3n-6 or 18:4n-3. **(C, F)** Mass spectra of unknown peaks (marked )? *Marks peaks shown on the main chromatogram and expanded insert for orientation only.

The amount of 18:3n-3 in stimulated cells was significantly (2.6-fold) greater after 48 h in medium containing an EFA ratio of 5:1 and 1.5-fold greater in medium containing an EFA ratio of 8:1 compared to unstimulated cells (**Table 2**). The amount of 20:5n-3 was significantly greater (1.5-fold) in stimulated than unstimulated cells after 48 h, but not at 14 or 24 h, in medium containing an EFA ratio of 5:1 but did not differ significantly between mitogen-stimulated and unstimulated cells maintained in medium with an EFA ratio of 8:1 (**Table 2**). There was no significant effect of mitogen stimulation on the amounts of 20:3n-3, 20:4n-3, 22:5n-6, or 22:6n-3 (**Table 2**). 18:4n-3 and 18:3n-6 were not detected by either GCFID or GCMS (**Figure 3**).

**Figure 4:**
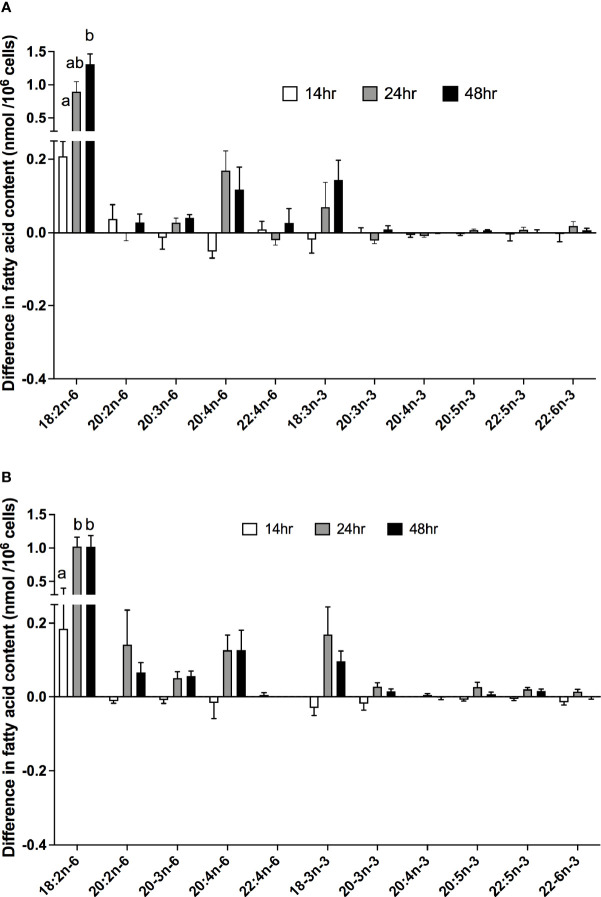
The effect of duration of incubation and essential fatty acid (EFA) ratio on the mitogen-induced change in the amounts of polyunsaturated fatty acids in CD3^+^ T cells. Values are mean ± SEM (n = 10/time point) difference in the amount of fatty acid in stimulated compared to unstimulated cells after 14-, 24-, and 48-h incubation in media containing an EFA ratio (18:2n-6:18:3n-3) of **(A)** 5:1 or **(B)** 8:1. Means represented by bars with different letters were significantly different by two-way ANOVA with Tukey’s *post-hoc* test. Results of statistical analyses are shown in **Table 4**.

There were significant effects of duration of incubation and the EFA ratio on the change in the amounts of individual PUFA in stimulated cells compared to unstimulated cells. For n-6 PUFA, the activation-induced change in the amount of 18:2n-6 increased by approximately 65-fold with greater incubation time [F(2,54) = 12.58, p < 0.0001], but there was no significant single-factor effect of the EFA ratio (p = 0.99) or time * EFA ratio interaction (p = 0.54) (**Figure 4A**). There were no significant single-factor effects of time (p = 0.22) or EFA ratio (p = 0.11) on the activation-induced change in the amount of 20:2n-6 (time, p = 0.43; EFA ratio, p = 0.23), 20:3n-6 (time, p = 0.72; EFA ratio, p = 0.54), 20:4n-6 (time, p = 0.09; EFA ratio, p = 0.99), or 22:4n-6 (time, p = 0.47; EFA ratio, p = 0.83).

For n-3 PUFA, there was a significant single-factor effect of time [F(2,54) = 5.32, p = 0.008), such that the activation-induced change in the amount of 18:3n-3 increased by approximately 8-fold between 14 and 24 h incubation, but there was no significant effect of EFA ratio (p = 0.74) or time * EFA ratio interaction (p = 0.34) (**Figure 4B**). The activation-induced change in the amount of 20:5n-3 increased by approximately 2-fold between 14 and 48 h incubation [time F(2,54) = 5.81, p = 0.005], but there was no significant effect of the EFA ratio (p = 0.32) or time * EFA ratio (p = 0.16) interaction. There were no significant single-factor effects of time or EFA ratio on the activation-induced change in the amount of 20:3n-3 (p = 0.18 and 0.21, respectively), 20:4n-3 (p = 0.92 and p = 0.08, respectively), 22:5n-3 (p = 0.09 and p = 0.24, respectively), and 22:6n-3 (p = 0.11 and p = 0.15, respectively).

### Effect of Mitogen Activation, Length of Incubation, and EFA Ratio on n-6 PUFA Synthesis in T Lymphocytes

There was no significant difference in the uptake of [d_5_]18:2n-6 between unesterified and ethyl-18:2n-6 in unstimulated (p = 0.9998) or stimulated T cells (p = 0.9993) (**Supplementary Figure S2**). There was no significant difference in the conversion of [d5]18:2n-6 to [d_5_]20:2n-6 between unstimulated (p = 0.1424) and stimulated T cells (p = 0.9993) (**Supplementary Figure S2**).

**Figure 5:**
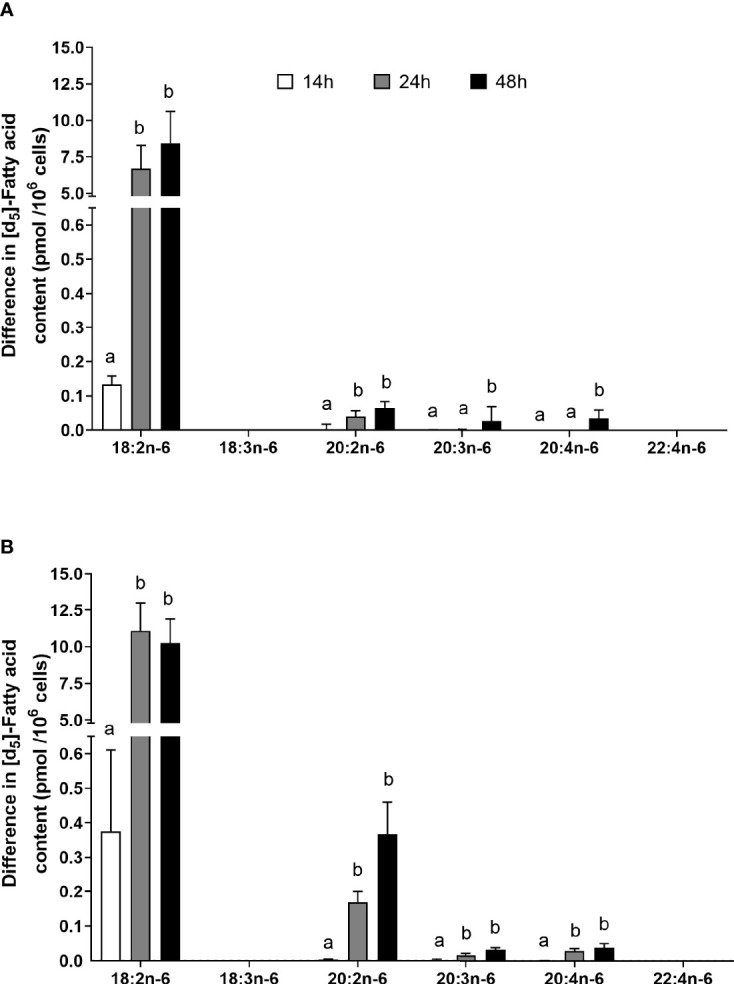
The effect of duration of incubation and essential fatty acid (EFA) ratio on the mitogen-induced change in the amount of [d_5_] n-6 polyunsaturated fatty acids (PUFAs) in CD3^+^ T cells. Values are mean ± SEM (n = 10/time point) change in the amount of labelled longer-chain PUFA (LCPUFA) after 14-, 24-, and 48-h incubation in media containing an EFA ratio (18:2n-6:18:3n-3) of **(A)** 5:1 or **(B)** 8:1. Means represented by bars with different letters were significantly different by two-way ANOVA with Tukey’s *post-hoc* test. Results of statistical analyses are shown in **Table 4**.

The amount of labelled PUFA in stimulated T cells was approximately 1/1,000th of the total amount of each PUFA (**Tables 2**, **3**). For the n-6 series, [d_5_]18:2n-6, [d_5_]20:2n-6, [d_5_]20:3n-6, and [d_5_]20:4n-6 were detected in unstimulated and stimulated T cells (**Table 3** and **Figure 5**). Here, [d_5_]18:2n-6 was the predominant labelled n-6 fatty acid at all time points measured regardless of the EFA ratio and cell activation (**Table 2**). Mitogen stimulation significantly increased the amount of [d_5_]18:2n-6 at 24 and 48 h by approximately 2-fold each regardless of the EFA ratio in the culture medium (**Table 3**). Mitogen stimulation increased the amount of the [d_5_]18:2n-6 elongation product [d_5_]20:2n-6 by 4.5-fold at 24 h and at 48 h regardless of the EFA ratio (**Table 3**). There was no significant effect of mitogen stimulation on the amount of [d_5_]20:3n-6 or [d_5_]20:4n-6 in cells cultured in medium containing an EFA ratio of 5:1 (**Table 3**). However, mitogen stimulation increased the amount of [d_5_]20:3n-6 (1.6-fold) and [d_5_]20:4n-6 (1.2-fold) in cells cultured in medium containing an EFA ratio of 8:1 for 48 h (**Figure 5** and **Table 4**).

**Table 3:** Effect of mitogen stimulation on T-cell PUFA biosynthesis.

Time	14 h			24 h			48 h		
	pmol/10^6^ cells		t-test	pmol/10^6^ cells		t-test	pmol/10^6^ cells		t-test
	U	S	adjP	U	S	adjP	U	S	adjP
	EFA ratio 5:1								
	[d_5_] n-6 series								
18:2n-6	13.47 ± 2.61	13.61 ± 0.84	0.962	6.262 ± 0.292	12.96 ± 1.53	0.020	9.60 ± 0.52	18.04 ± 2. 6	0.036
20:2n-6	0.03 ± 0.02	0.03 ± 0.01	0.799	0.011 ± 0.003	0.05 ± 0.02	0.972	0.02 ± 0.01	0.09 ± 0.02	0.013
20:3n-6	0.01 ± 0.01	0.016 ± 0.01	0.511	0.012 ± 0.001	0.01 ± 0.01	0.799	0.04 ± 0.01	0.12 ± 0.05	0.501
20:4n-6	0.03 ± 0.04	0.017 ± 0.01	0.163	0.030 ± 0.004	0.02 ± 0.01	0.323	0.03 ± 0.01	0.07 ± 0.02	0.501
	[^13^C] n-3 series								
18:3n-3	6.78 ± 1.38	7.09 ± 0.43	0.834	4.31 ± 0.68	7.94 ± 0.76	0.004	5.76 ± 0.28	12.91 ± 1.30	<0.001
20:3n-3	0.11 ± 0.03	0.18 ± 0.05	0.260	0.14 ± 0.04	0.14 ± 0.02	0.253	0.11 ± 0.03	0.26 ± 0.08	0.337
20:5n-3	0.01 ± 0.01	0.01 ± 0.00	0.997	0.01 ± 0.00	0.01 ± 0.01	0.622	0.00 ± 0.00	0.02 ± 0.01	0.147
	EFA ratio 8:1								
	[d_5_] n-6 series								
18:2n-6	6.37 ± 1.15	6.0 ± 0.06	1.00	11.15 ± 0.79	22.48 ± 2.35	0.003	17.44 ± 1.81	27.69 ± 1.87	0.007
20:2n-6	0.006 ± 0.01	0.003 ± 0.001	0.47	0.18 ± 0.03	0.35 ± 0.04	0.019	0.24 ± 0.02	0.61 ± 0.09	0.007
20:3n-6	0.004 ± 0.01	0.003 ± 0.003	>0.9	0.05 ± 0.01	0.06 ± 0.01	0.026	0.05 ± 0.01	0.08 ± 0.01	0.002
20:4n-6	0.023 ± 0.02	0.02 ± 0.01	1.00	0.16 ± 0.01	0.19 ± 0.01	0.029	0.21 ± 0.01	0.25 ± 0.011	0.026
	[^13^C] n-3 series								
18:3n-3	3.17 ± 0.19	3.16 ± 0.41	1.00	3.21 ± 0.61	6.14 ± 0.82	0.036	7.88 ± 1.38	15.25 ± 2.02	0.044
20:3n-3	0.01 ± 0.00	0.01 ± 0.00	>0.9	0.01 ± 0.01	0.01 ± 0.01	0.798	0.02 ± 0.01	0.03 ± 0.01	0.232
20:5n-3	0.03 ± 0.01	0.02 ± 0.01	1.00	0.05 ± 0.02	0.13 ± 0.06	0.698	0.02 ± 0.00	0.03 ± 0.01	0.591

Values are mean ± SEM (n = 10 paired samples at each time point). Comparison between unstimulated and stimulated cells were done by Student’s paired t-test, and statistical significance was assumed at p < 0.05. Adjustment for multiple t-tests was by the Holm–Sidak method (adjP). EFA ratio (18:2n-6:18:3n-3 in the culture medium); S, mitogen-stimulated cells; U, unstimulated cells; EFA, essential fatty acid; PUFA, polyunsaturated fatty acid.

**Table 4:** Statistical analysis of the effects of EFA ratio and duration of incubation on the mitogen-induced change in the amount of labelled PUFA in T cells.

	Two-way ANOVA					
	Time		EFA ratio		Time * EFA ratio	
	F	p	F	p	F	p
[d_5_]18:2n-6	6.20	<0.001	1.3	0.3	30.1	0.50
[d_5_]20:2n-6	13.5	<0.001	17.3	<0.001	6.8	0.002
[d_5_]20:3n-6	4.86	0.012	0.40	0.53	1.87	0.25
[d_5_]20:4n-6	5.73	0.006	1.2	0.40	1.8	0.17
[^13^C]18:3n-3	19.62	<0.001	0.08	0.80	0.08	0.92
[^13^C]20:3n-3	5.69	0.02	3.54	0.03	1.47	0.24
[^13^C]20:5n-3	2.48	0.09	1.74	0.19	2.07	0.14

Values were calculated using two-way ANOVA with time and EFA ratio as fixed factors. Post-hoc pairwise comparisons between time points within an EFA ratio were done by Tukey’s test. Means that differed significantly (p < 0.05) are indicated by different superscripts. Degrees of freedom were 1,60. ηp^2^ was ≥0.14 for all statistically significant outcomes. EFA, essential fatty acid; PUFA, polyunsaturated fatty acid.

There was a significant effect of time but no single-factor effect of the EFA ratio nor time * EFA ratio interaction, such that the activation-induced change in the amount of [d_5_]18:2n-6, [d_5_]20:3n-6, and [d_5_]20:4n-6 increased by 63-fold, 7.5-fold, and 4.1-fold between 14 and 48 h, respectively (**Table 3**). There were significant single-factor effects of time and EFA ratio and a significant time * EFA ratio interaction on the activation-induced change in the amount of [d_5_]20:2n-6 (**Table 3**). The activation-induced change in the amount of [d_5_]20:2n-6 increased approximately 65-fold between 14 and 24 h in cells maintained in medium containing an EFA ratio of 5:1 and 185-fold in cells cultured in medium with an EFA ratio of 8:1 (**Figure 5** and **Table 4**).

### Effect of Mitogen Activation, Length of Incubation, and EFA Ratio on n-3 PUFA Synthesis in T Lymphocytes

**Figure 6:**
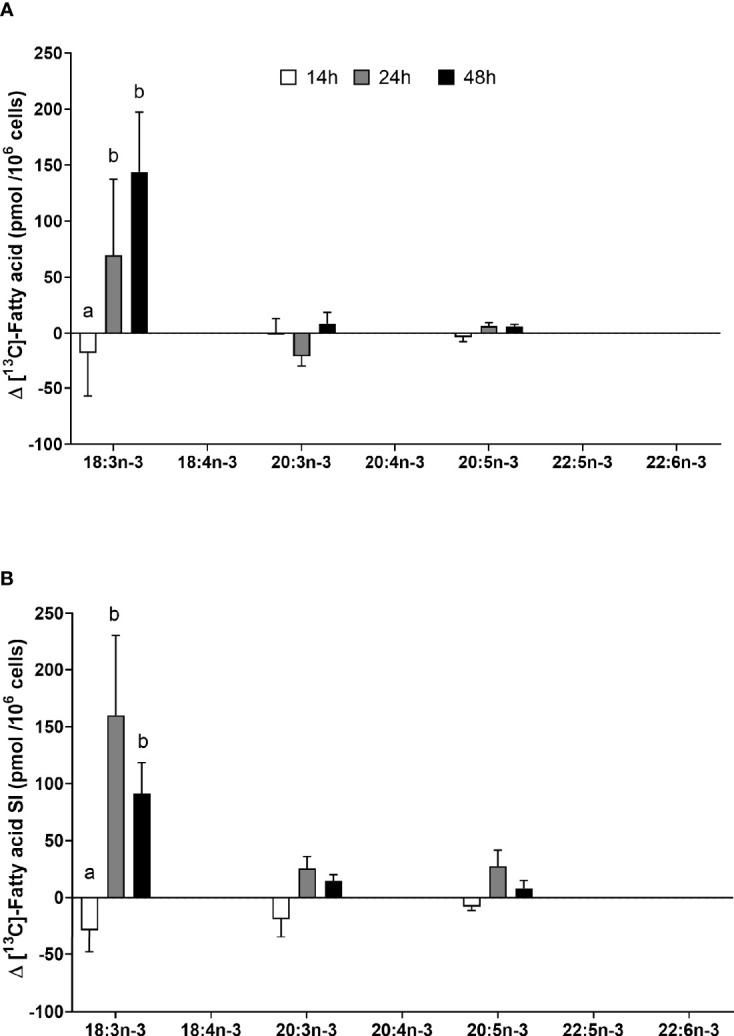
The effect of duration of incubation and essential fatty acid (EFA) ratio on the mitogen-induced change in the amount of [^13^C] n-3 polyunsaturated fatty acids (PUFAs) in CD3^+^ T cells. Values are mean ± SEM (n = 10/time point) change in the amount of labelled longer-chain PUFA (LCPUFA) after 14-, 24-, and 48-h incubation in media containing an EFA ratio of **(A)** 5:1 or **(B)** 8:1. Means represented by bars with different letters were significantly different by two-way ANOVA with Tukey’s *post-hoc* test (**Table 4**).

[^13^C]18:3n-3 was the predominant labelled n-3 fatty acid at all time points measured regardless of the EFA ratio and activation state (**Table 3**). Here, [^13^C]18:3n-3, [^13^C]20:3n-3, and [^13^C]20:5n-3 were detected in T cells regardless of the activation state (**Table 3**). Mitogen stimulation significantly increased the amount of [^13^C]18:3n-3 at 24 and 48 h, but not at 14 h, regardless of the EFA ratio in the culture medium (**Table 3** and **Figure 6**). There was no significant effect of mitogen stimulation on the amount of [^13^C]20:3n-3 at any time point measured regardless of the EFA ratio in the culture medium (**Table 3**). However, mitogen stimulation increased the amount of [^13^C]20:5n-3 above that of unstimulated cells cultured in medium with an EFA ratio of 5:1 at 48 h, but there was no significant effect of mitogen stimulation on the amount of [^13^C]20:5n-3 in cells cultured in medium containing an EFA ratio of 8:1 (**Figure 6** and **Table 4**).

There were significant single-factor effects of time and EFA ratio on activation-induced change in the amount of [^13^C]20:3n-3 and of time on the activation-induced change in the amount of [^13^C]20:5n-3 (**Figure 6** and **Table 3**). [^13^C]18:4n-3, [^13^C]20:4n-3, [^13^C]22:5n-3, and [^13^C]22:6n-3 were not detected after 48-h culture (**Tables 3**, **4**).

### The Effect of Mitogen Activation and EFA Ratio on Oxylipin Synthesis in T Lymphocytes

**Figure 7:**
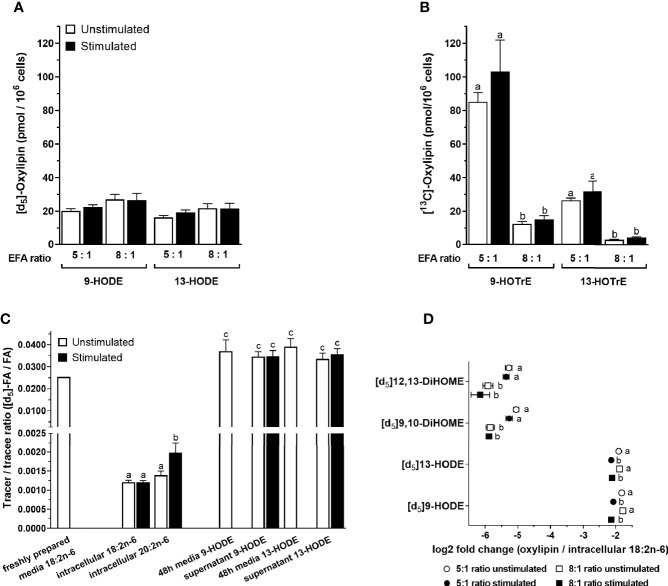
The effect of essential fatty acid (EFA) ratio and mitogen stimulation on the concentrations of [^13^C]18:3n-3 and [d_5_]18:2n-6-derived oxylipins in culture supernatants after 48 h. LC-MS/MS analysis of labelled oxylipins in the supernatant from 48-h cultured human CD3^+^ T cells with an EFA ratio of either 5:1 or 8:1. Statistical analysis was performed in SPSS with two-way paired ANOVA **(A, B, D)** and **(C)** one-way ANOVA with Sidak’s unpaired multiple comparisons of individual oxylipins. Different letters mark significant changes between stimulation, EFA ratio, and tracer-to-tracee ratio within individual oxylipins. **(A)** Here, [d_5_]18:2n-6 oxidation products 9- and 13-HODE were not altered by either EFA ratio or stimulation. **(B)** In addition, [1-^13^C]18:3n-3 oxidation products 9- and 13-HOTrE increase with higher 18:3n-3 concentration in 5:1 EFA ratio. **(C)** Tracer (labelled)-to-tracee (unlabelled) ratios were calculated for [d_5_]HODE with an 8:1 ratio of 18:2n-6/18:3n-3 and compared to intracellular [d_5_]18:2n-6, elongation product [d_5_]20:2n-6, and non-enzymatically oxidised [d_5_]HODE in freshly prepared and 48-h cultured cell media (in absence of T cells). **(D)** Here, [d_5_]HODE and its oxidation product [d_5_]DiHOME normalised to intracellular [d_5_]18:2n-6 (GC-IRMS) are shown as log2 fold change.

[^13^C]9-Hydroxyoctadecatrienoic acid ([^13^C]9-HOTrE) was the most abundant of the labelled oxylipins that were measured in the T-cell culture supernatant containing an EFA ratio of 5:1, followed by lower concentrations of 9-HODE, 13-HODE, 13-HOTrE, 9,10-DiHOME, 12,13-DiHOME, 9,10-DiHODE, 12,13-DiHODE, and 15,16-DiHODE (**Figures 7A, B** and **Supplementary Figure S3**). There were no significant single-factor or interaction effects of cell stimulation and EFA ratio on the concentrations of either [d_5_]9-HODE (stimulation, p = 0.461; EFA ratio, p = 0.129) or [d_5_]13-HODE (stimulation, p = 0.177; EFA ratio, p = 0.205) in the T-cell supernatant (**Figure 7A**). However, [d_5_]9,10-DiHOME concentration was 3.2-fold greater [F(1,18) = 13.65, p = 0.002] in the supernatant from stimulated cultures with an EFA ratio of 5:1 compared to 8:1 (**Supplementary Figure S3A**). There were no significant single-factor or interaction effects of cell activation on [d_5_]9,10-DiHOME. Here, [d_5_]12,13-DiHOME concentration in the culture supernatant with an EFA ratio of 5:1 from stimulated cells was 1.6-fold [F(1,18) = 5.53, p = 0.03] greater than from unstimulated cultures. In addition, [d_5_]12,13-DiHOME concentration in supernatants with an EFA ratio of 5:1 from mitogen-stimulated cells was 3.3-fold greater [F(1,18) = 14.66, p = 0.001] than supernatants with an EFA ratio of 8:1 (**Supplementary Figure S2A**).

There were no significant single-factor effects of EFA ratio on [^13^C]9-HOTrE, [^13^C]13-HOTrE, or DiHODE concentrations in the culture supernatants (**Figure 7B** and **Supplementary Figure S2B**). Here, [^13^C]9-HOTrE concentration was 6.8-fold greater [F(1,18) = 79.99, p < 0.001] in supernatants from cultures with an EFA ratio of 5:1 than 8:1. A similar effect was seen in the [^13^C]13-HOTrE concentration with a 7.5-fold increase [F(1,18) = 71.18, p < 0.001] in supernatants from cultures with an EFA ratio of 5:1 compared to 8:1. DiHODE concentrations were significantly greater in cultures with an EFA ratio of 5:1 than 8:1 [F(1,18) = 78.66, p < 0.001; **Supplementary Figure S3B**].

The tracer-to-tracee ratio (TTR) was calculated for [d_5_]18:2n-6 and its metabolites, namely, [d_5_]20:2n-6, [d_5_]9-HODE, and [d_5_]13-HODE, to investigate the relative contribution of recently internalised EFA compared to the preexisting EFA pools in T cells to the synthesis of LCPUFA and oxylipins (**Figure 7C**). Freshly prepared cell media with an EFA ratio of 8:1 and containing [d_5_]18:2n-6 tracer that had been stored at 4°C had a TTR of 0.0255. In contrast, intracellular [d_5_]18:2n-6 from activated T cells collected after 48-h incubation had a TTR of 0.0012% ± 4%. This is equivalent to a 20-fold dilution of [d_5_]18:2n-6 by endogenous unlabelled 18:2n-6. Intracellular [d_5_]20:2n-6 had a 1.65-fold (p = 0.049; Sidak’s paired multiple comparison) higher TTR (0.00198 ± 14%) compared to [d_5_]18:2n-6 after 48-h stimulated cell culture.

Culture media with an EFA ratio of 8:1 and containing the [d_5_]18:2n-6 tracer that had been incubated at 37°C in the absence of cells had detectable amounts of (non-enzymatically) oxidised [d_5_]9-HODE and [d_5_]13-HODE with TTR values of 0.0372 ± 13% and 0.0391 ± 9%, respectively. The TTR of [d_5_]9-HODE and [d_5_]13-HODE in the supernatant from T-cell cultures incubated for 48 h were 0.0347 ± 8% and 0.0358 ± 7%, respectively, which was similar to the TTR of HODEs in 48-h cultured media without cells (p > 0.05; Sidak’s unpaired multiple comparisons) but higher than recently internalised 18:2n-6 (p < 0.001) (**Figure 7C**). Non-enzymatically formed 9-HODE in the control culture media (n = 3, 12 nmol/L) accounted for approximately one-third of total 9-HODE quantified in the 48-h T-cell supernatant and represents therefore a significant difference in concentrations [n = 10, 39 nmol/L; one-way ANOVA F(4,28) = 24.4, p = 0.002] (**Supplementary Figure S4**).

The amount of [d_5_]HODEs and its dihydroxy metabolite, namely, [d_5_]DiHOME, was normalised to the intracellular [d_5_]18:2n-6 substrate pool (**Table 3**) and displayed as log2 fold change (oxylipin/18:2n-6). There was a significant effect of stimulation on the change in [d_5_]9-HODE [F(1,18) = 56.13, p < 0.001] and [d_5_]13-HODE [F(1,18) = 34.82, p < 0.001] concentrations relative to the 18:2n-6 pool in the supernatants from 48-h T-cell cultures for both EFA ratios. There was no significant effect of the EFA ratio on the change in the concentrations of [d_5_]9-HODE [F(1,18) = 0.34, p = 0.569] and [d_5_]13-HODE [F(1,18) = 0.10, p = 0.754] in supernatants from unstimulated and stimulated cells (**Figure 7D**). There was no significant effect of stimulation on the change in [d_5_]9,10-DiHOME (p = 0.1) and [d_5_]12,13-DiHOME (p = 0.41) concentrations, while there was a significant effect of EFA ratio on the change in [d_5_]9,10-DiHOME [F(1,18) = 34.25, p < 0.001] and [d_5_]12,13-DiHOME [F(1,18) = 19.77, p < 0.001] (**Figure 7D**) concentrations.

## Discussion

The findings show that mitogen stimulation of purified human CD3^+^ T lymphocytes induced modest changes in total cell fatty acid composition, specifically, increased amounts of monounsaturated fatty acids, EFA, and longer-chain n-6 PUFA. These changes were accompanied by increased conversion of [d_5_]18:2n-6 and [^13^C]18:3n-3 to LCPUFA *via* a pathway consistent with EFA elongation followed by Δ8 desaturation of the primary product (34) and synthesis and secretion into the supernatant of 9- and 13- [d_5_]HODE and 9- and 13- [^13^C]HOTrE.

Previous studies show that mitogen activation involves selective changes in the fatty acid composition of human T lymphocytes, primarily increased proportions of 18:1n-9, 22:5n-3, and 22:6n-3 and decreased amounts of 18:2n-6 and 20:4n-6 over a period of up to 144 h (22), although this process may be faster in cells from other animal species (23). In contrast, the current findings did not show any significant effect of mitogen stimulation on the amounts of 22:5n-3 or 22:6n-3, while the amounts of 18:2n-6 and 20:4n-6 were greater in stimulated than unstimulated cells, which is in general agreement with findings reported previously (35). Similar to one previous report (22), the present findings showed that the amount of 18:1n-9 was significantly greater in stimulated compared to unstimulated cells. Differences between studies may be due to the manner in which the data were presented, specifically, proportions of total fatty acids (22) compared to reporting the amounts of individual fatty acids per million cells used here and previously (35). The latter approach was used in the present study in order to compare directly the pattern of newly synthesised fatty acids derived from stable isotope tracers with activation-associated changes in total cell lipids.

Previous studies using tracers in PBMCs and analysis of changes in fatty acid composition (35) and measurement of enzyme activities in isolated T cells (31) show that mitogen stimulation increased uptake of EFA substrates and induced Δ9-, Δ6-, and Δ5-desaturase activities (31). One study using stable isotope tracers in PBMCs showed that mitogen stimulation induced conversion of EFA to LCPUFA (34). The initial reactions were carbon chain elongation, possibly by elongase-5 activity (35), followed by Δ8 desaturation that has been suggested to be catalysed by the protein product of *FADS2* known as Δ6 desaturase (34). This is supported by the findings that the *FADS2* protein can exhibit both Δ6 and Δ8 desaturase activities (36) and that a single *FADS2* transcript corresponding to the predominant isoform is expressed in PBMCs and in Jurkat T lymphocyte leukaemia cells that show Δ6 and Δ8 desaturase activities (34). The present study failed to detect conversion of 18:2n-6 to 18:3n-6 or 18:3n-3 to 18:4n-3, and neither 18:3n-6 nor 18:4n-3 was present in stimulated or unstimulated cells. Instead, the main products of [d_5_]18:2n-6 interconversion were 20:2n-6, 20:3n-6, and 20:4n-6 and those of [^13^C]18:3n-3 interconversion were 20:3n-3 and 20:5n-3, which is consistent with initial carbon chain elongation followed by Δ8 and Δ5 desaturation. Here, [^13^C]20:4n-3 could not be quantified. These findings support the view that the first two reactions of the PUFA synthesis pathway in T cells are reversed compared to the well-characterised hepatic PUFA synthesis pathway (29, 30). There was no evidence of conversion of 18:2n-6 or 18:3n-3 to 22-carbon PUFA. This is consistent with the absence of *ELOVL2* expression, which is in agreement with previous reports in quiescent and mitogen-activated PBMCs (34) and human T cells (35). Moreover, mitogen stimulation of PBMCs has been reported to increase *ELOVL4* mRNA expression (34). However, the present study did not detect *ELOVL4* expression in unstimulated or stimulated T cells or stable isotope enrichment of PUFA >28 carbons long (data not shown). One possible explanation is that elongase-4 is expressed in other cell types present in the PBMC preparation but absent from purified T lymphocytes.

The time course of changes in PUFA synthesis following activation of T cells has not been reported previously. The present findings show that mitogen stimulation induced increased cell surface expression of CD69 and upregulation of *FADS2* mRNA expression that significantly increased compared to unstimulated cells after 14 h, while differential expression of *FADS1* was not detected until 24 h after stimulation. However, *ELOVL5* expression did not change significantly in stimulated cells from that in unstimulated cells at any time point measured. One interpretation is that increased capacity for conversion of EFA to LCPUFA is not a feature of early T-cell activation since the amount of 20-carbon PUFA in stimulated cells did not exceed that in unstimulated cells until 48 h after activation, which is later in the immune response than the onset of changes in membrane fatty acid composition (20). Furthermore, capacity for PUFA synthesis may be limited by the expression of *FADS* 1 and 2. However, the capacity for chain elongation *via* elongase-5 activity, the putative catalyst of the first reaction in the T-cell PUFA synthesis pathway (35, 52), in unstimulated T cells may be sufficient to support PUFA synthesis in stimulated cells without increased expression of the ELOVL5 transcript.

Here, [^13^C]18:3n-3 and [d_5_]18:2n-6 accumulation was greater in stimulated than unstimulated cells at 24 and 48 h after activation, but not at 14 h, which is consistent with the findings of previous studies of the effect of mitogen stimulation for 24 h on the fatty acid composition of T cells incubated with EFA. In addition, [d_5_]18:2n-6 accumulation was greater in cells maintained in media with an EFA ratio of 8:1 than those incubated in media with an EFA ratio of 5:1. However, the EFA ratio did not appear to affect [^13^C]18:3n-3 accumulation. This suggests selectivity in stimulated T cells that has not been noted previously (24). One possible implication is that the availability of EFA substrates could influence flux through the PUFA synthesis pathway T cells.

Differences in the relative dietary intakes of 18:3n-3 and 18:2n-6 can alter the flux of n-3 and n-6 through the hepatic PUFA synthesis pathway in rodents (53, 54) and humans (55). The present findings show that despite reversal of the first two reactions in T cells compared to the liver, the EFA ratio modified conversion of EFA to LCPUFA in the same manner; specifically, a higher ratio of 18:2n-6 to 18:3n-3 (8:1) reduced conversion of 18:3n-3 to LCPUFA accompanied by greater conversion of 18:2n-6. Elongase-5 can elongate PUFAs that are 16–20 carbons long. However, competition between 18:2n-6 and 18:3n-3 for elongase-5 activity has not been reported. Therefore, it is possible that, as in the liver (53), competition for FADS2 protein activity mediates the effect of the EFA ratio on PUFA synthesis in T cells. The range of daily intakes of 18:2n-6 and 18:3n-3 has been reported to differ between countries by approximately 4-fold (37). Whether such nutritional trends acting *via* the capacity for PUFA synthesis in T cells contribute to patterns of inflammatory or allergic disease remains to be investigated.

Overall, these findings show that the pattern of newly synthesised PUFA differs from mitogen-induced changes in T-cell total fatty acid composition. This suggests that the primary function of PUFA synthesis in T cells is not to provide substrates for membrane synthesis that agrees with the view of Anel et al. (31) that mitogen-induced changes in desaturase activities are insufficient to explain the adaptations to membrane fatty acid composition associated with blastogenesis. Moreover, changes to the fatty acid composition of lymphocyte membrane phospholipids during blastogenesis have been shown to reflect changes in the specificity of phospholipid biosynthesis, in particular, altered activities of acyl-remodelling mechanisms (23, 24). One further implication is that product-to-precursor ratios of cell total fatty acids are not an appropriate proxy measure of desaturase or elongase activities in T lymphocytes.

Pharmacological inhibition of *FADS2* protein activity reduced T-cell proliferation (34), although others failed to detect an effect of partial *ELOVL5* knockdown on T-cell activation or apoptosis (35). One possible interpretation is that if *FADS2* activity is limiting, but not elongase-5, which is suggested by the present findings, then inhibition of the *FADS2* protein activity is likely to have a greater effect on the regulation of T-cell function by PUFA biosynthesis than partial knockdown of *ELOVL5*.

The present findings show that [d_5_]18:2n-6 and [^13^C]18:3n-3 were oxidised to 9- and 13-HODE, and 9- and 13-HOTrE, respectively, probably by lipoxygenase activity (12). Alternatively, [d_5_]18:2n-6 and [^13^C]18:3n-3 were di-hydroxylated to 9,10- and 12,13-DiHOME, and 9,10-, 12,13-, and 15,16-DiHODE, probably by cytochrome p450 activity (13). These 18-carbon oxylipins were secreted into the culture supernatant of both unstimulated and stimulated T cells. Although accumulation of [d_5_]18:2n-6 by stimulated T cells was greater than that of [^13^C]18:3n-3, the concentrations of [^13^C]9-HOTrE and [^13^C]13- HOTrE were greater than those of [d_5_]9-HODE and [d_5_]13-HODE, which suggests preferential partitioning of 18:3n-3 towards oxidation. If so, preferential HOTrE synthesis may contribute to the lower amounts of 18:3n-3-derived LCPUFA than those from 18:2n-6. Hydroxyoctadecaenoic acids appeared to be formed preferentially from recently internalised [d_5_]18:2n-6 compared to the preexisting endogenous 18:2n-6 pool that implies partitioning towards lipoxygenase activity may be an early event in T-cell EFA metabolism. Furthermore, mitogen stimulation decreased the fold change of HODEs relative to 18:2n-6 in T cells, while the fold change of DiHOMEs relative to 18:2n-6 was not affected, suggesting independent regulation of 18:2n-6 conversion into oxylipins by lipoxygenase and cytochrome P450 enzymes.

Newly assimilated EFAs were preferentially used for synthesis of hydroxyoctadecaenoic and dihydroxyoctadecaenoic acids, presumably by lipoxygenase and cytochrome P450 activities that appear to be regulated independently. Since labelled 20-carbon oxylipins were not detected, these findings are consistent with preferential partitioning of EFA to 18-carbon oxylipins in activated T cells. The functions of 18:2n-6 and 18:3n-3 derived oxylipins are less well characterised than those formed from LCPUFA, namely, eicosanoids and specialised pro-resolving mediators (12, 56). Both 9- and 13-HOTrE have been reported to induce glomerular hypertrophy and 13-HOTrE to suppress interleukin-1β action, while 9- and 13-HODE are antiproliferative (12, 56). 9-HODE has been shown to be pro-inflammatory, while 13-HODE can have anti-thrombotic and anti-inflammatory actions (12). There is some evidence that DiHOMEs can induce a range of biological effects (13). For example, 9,10-DiHOME can induce both enhanced and impaired neutrophil chemotaxis, depending on the concentration of the dihydroxy-metabolite (57, 58), while 12,13-DiHOME has been associated with acute lipaemic-induced inflammation (59). However, the precise function of HODEs and HOTrEs in T cells has yet to be described but may represent novel mediators in the regulation of T lymphocyte activation that can be modified by dietary lipids.

**Figure 8:**
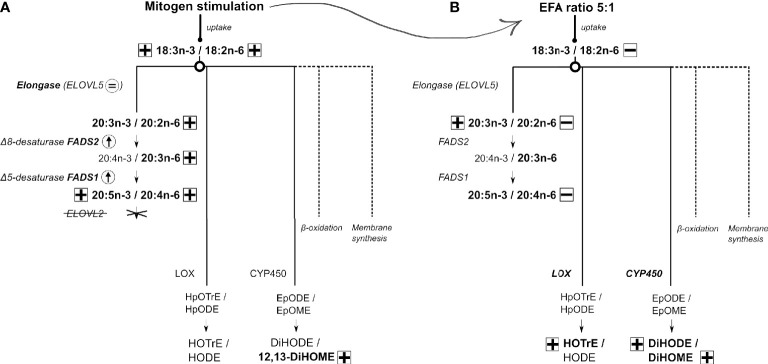
A model for 18:3n-3 and 18:2n-6 metabolism in human CD3^+^ T lymphocytes and the effect of response to stimulation and different ratios of 18:3n-3 and 18:2n-6 substrates. Arrow or an equal sign within a circle indicates either an increase or no change in mRNA expression. Plus or minus in a square indicates either an increase or decrease in polyunsaturated fatty acid (PUFA)/oxylipin concentration, respectively. Dotted lines refer to possible other mechanisms not further explored in this work. **(A)** The metabolic fates of 18:3n-3 and 18:2n-6 in stimulated compared to unstimulated T cells. **(B)** The metabolic fates of 18:3n-3 and 18:2n-6 in stimulated cells cultured in medium with a 18:2n-6:18:3n-3 ratio of 8:1 compared to cells maintained in medium with a 18:2n-6:18:3n-3 ratio of 5:1. A detailed description is presented in the *Discussion*.

Based on these findings, we suggest the following model of EFA metabolism in T lymphocytes (**Figure 8**). Stimulation of T cells increases the uptake of EFA by a mechanism that is influenced by the relative amounts of EFA substrates in the extracellular environment. It is not known whether this reflects selectivity by fatty acid transporters and/or competition between n-6 and n-3 EFA. Newly assimilated EFA may then be partitioned towards beta-oxidation (60), which can contribute 50%–90% of ATP synthesis in leukocytes (61), and membrane synthesis to support programmed changes in T-cell membrane fatty acid composition that are associated with blastogenesis (19–21, 23, 24), synthesis of LCPUFA, or enzymatic oxidation to form oxylipins. The present findings show that partitioning between oxylipin synthesis and conversion to LCPUFA is a branch point in EFA metabolism, although differential distribution between the remaining fates cannot be deduced from these data. In contrast to the liver, the PUFA synthesis pathway in T cells is limited to the synthesis of 20-carbon PUFA by the absence of elongase-2 expression that appear to be derived preferentially from recently internalised EFA. Therefore, the synthesis of LCPUFA from EFA appears unlikely to be a primary source of substrates for activation-induced remodelling of lymphocyte membranes. One possible explanation is that conversion of EFA to a restricted number of LCPUFA may facilitate partitioning of EFA towards alternative pathways. If so, product inhibition of LCPUFA synthesis by dietary supplementation with 20:5n-3 and 22:6n-3 (62) could further potentiate oxylipin synthesis and so represent a novel mechanism in the immunomodulatory action of fish oil. Moreover, modulation of differential partitioning of EFAs by the ratio of 18:2n-6 to 18:3n-3 could contribute to the pro-inflammatory effects attributed to some dietary patterns (63).

## Data Availability Statement

The raw data supporting the conclusions of this article will be made available by the authors without undue reservation.

## Ethics Statement

The studies involving human participants were reviewed and approved by East of England-Cambridge Central Research Ethics Committee (approval number 19/EE/0096). The participants provided their written informed consent to take part in this study.

## Author Contributions

GB, BF, PC, EM, and KL conceived and designed the study. JG, AW, and NI carried out the experiments and, together with GB, analysed the data. GB wrote the first draft of the article with inputs from all authors. All authors contributed to the article and approved the submitted version.

## Funding

This research was supported by grants from the Biological Sciences and Biotechnology Research Council (grant numbers BB/S00548X/1 and BB/S005358/1). The funder was not involved in the study design, collection, analysis, interpretation of data, the writing of this article, or the decision to submit it for publication. Publication costs were paid by the University of Southampton.

## Conflict of Interest

GB has received research funding from Nestle, Abbott Nutrition, and Danone and has served as a member of the Scientific Advisory Board of BASF. PC acts as a consultant to BASF, Smartfish, DSM, Cargill, Danone/Nutricia, and Fresenius-Kabi. KL has received research funding from Nestle, Abbott Nutrition, and Danone.

The remaining authors declare that the research was conducted in the absence of any commercial or financial relationships that could be construed as a potential conflict of interest.

## Publisher’s Note

All claims expressed in this article are solely those of the authors and do not necessarily represent those of their affiliated organizations, or those of the publisher, the editors and the reviewers. Any product that may be evaluated in this article, or claim that may be made by its manufacturer, is not guaranteed or endorsed by the publisher.
