# Supplementary material for: The Partitioning of Newly Assimilated Linoleic and α-Linolenic Acids Between Synthesis of Longer-Chain Polyunsaturated Fatty Acids and Hydroxyoctadecaenoic Acids Is a Putative Branch Point in T-Cell Essential Fatty Acid Metabolism
Source: Front Immunol. 2021 Oct 5;12:740749. doi: 10.3389/fimmu.2021.740749 (PMC8523940; doi:10.3389/fimmu.2021.740749)
Supplement: Supplementary file 1 [file DataSheet_1.pdf]

Supplementary online material

Supplementary Table 1 Oxylipin multiple reaction monitoring transitions for LC-MS/MS analysis.

Oxylipin	Q1 $[M-H]^-$	Q2 [m/z]	Tracer	Q1 $[M-H]^-$	Q2 [m/z]	CE [eV]
[d_5]17-HDHA	m/z 348.3	201<br>286	Internal standard			15
9-HODE	m/z 295.2	171.1<br>233.2	[d_5]9-HODE	m/z 300.3	171.1<br>238.3	25
13-HODE	m/z 295.2	195.2<br>251.2	[d_5]13-HODE	m/z 300.3	195.2<br>256.3	25

Supplementary Table 2 Real-time RTPCR primers.

**Primer target**	**Primer Assay**
FADS1	Qiagen Hs_FADS1_2_SG Quantitect Primer Assay (QT02322621)
FADS2	Qiagen Hs_FADS2_1_SG Quantitect Primer Assay (QT00077175)
ELOVL2	Qiagen Hs_ELOVL2_1_SG Quantitect Primer Assay (QT00059017)
ELOVL4	Qiagen Hs_ELOVL4_1_SG Quantitect Primer Assay (QT00017283)
ELOVL5	Qiagen Hs_ELOVL5_1_SG Quantitect Primer Assay (QT00096334)
RPL13A	Primer design reference gene assay (HK-SY-hu)
SDHA	Primer design reference gene assay (HK-SY-hu)

Supplementary Figure 1 LC-MS/MS validation of the chromatographic method and internal standard.

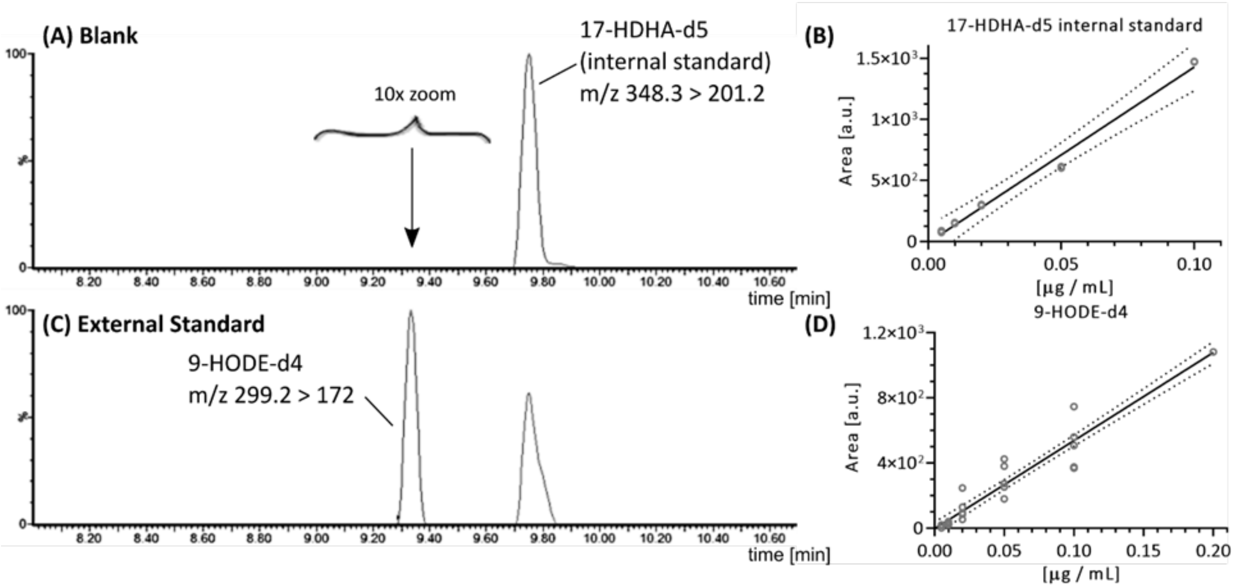

(A) LC-MS/MS chromatogram of internal standard [d_5]17-HDHA showing no peak at the expected retention time of 9-HODE (blank). Calibration curve for limit of detection (LOD) determination of (B) [d_5]17-HDHA and (D) [d_4]9-HODE. (C) Chromatographic separation of external standard [d_4]9-HODE at the expected retention time and internal standard [d_5]17-HDHA.

Supplementary Figure 2 Comparison of the uptake and conversion of ethyl-[d5]18:2n-6 to 18:2n-6 in unstimulated and mitogen stimulatedT cells.

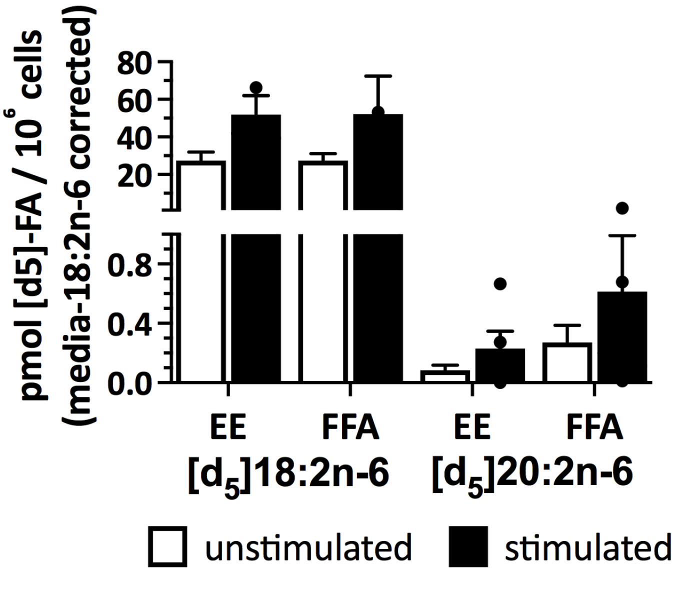

Values are mean ± SEM (n = 5 cultures per molecular form for unstimulated or stimulated T cells). Symbols indicate individual outlying data points. Statistical comparisons between molecular forms were by Two-Way ANOVA with Holm-Šídák's multiple comparisons test. There were no statitically significant differences in uptake between ethyl ester (EE) and free fatty acid (FFA) forms of [d_5]18:2n-6, or in conversion to [d_5]20:2n-6.

Supplementary Figure 3 Effect of mitogen stimulation and EFA ratio on [d_5]18:2n-6 and [1-^{13}C]18:3n-3 oxidation products DiHOME and DiHODE.

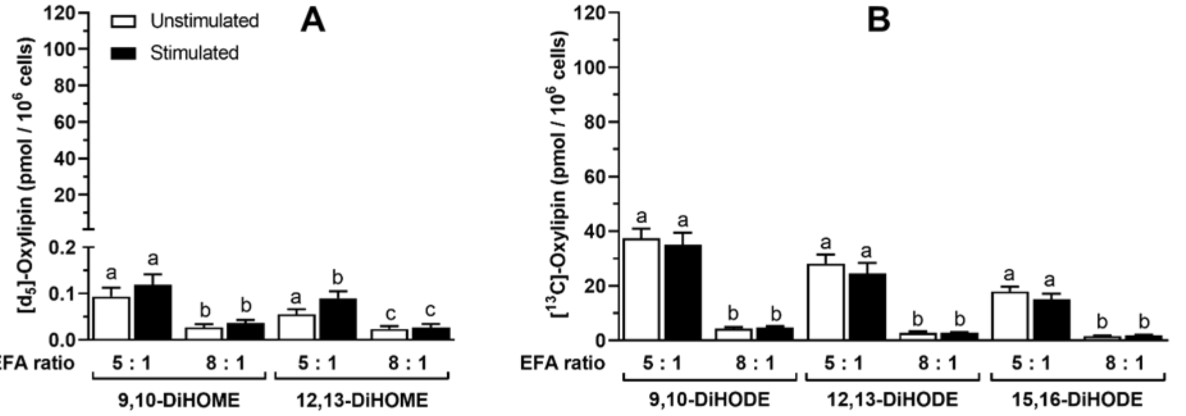

LC-MS/MS analysis of labelled oxylipins in the supernatant of 48h cultured human CD3+ T cells with an EFA ratio of either 5:1 or 8:1. Statistic analysis was performed in SPSS with 2-way paired ANOVA for individual oxylipins. Different letters mark significant changes between stimulation and EFA ratio within individual oxylipins. (A) [d_5]18:2n-6 oxidation products 9,10- and 12,13-DiHOME change with EFA ratio and only 9,10-DiHOME with stimulation. (B) [1-^{13}C]18:3n-3 oxidation products 9,10- , 12,13- and 15,16-DiHODE increase with higher 18:3n-3 concentration in 5:1 EFA ratio.

Supplementary Figure 4 Total 9-HODE and tracer [d_5]9-HODE determination in culture media controls, T cell control without EFA added and T cells with an EFA ratio of 8:1.

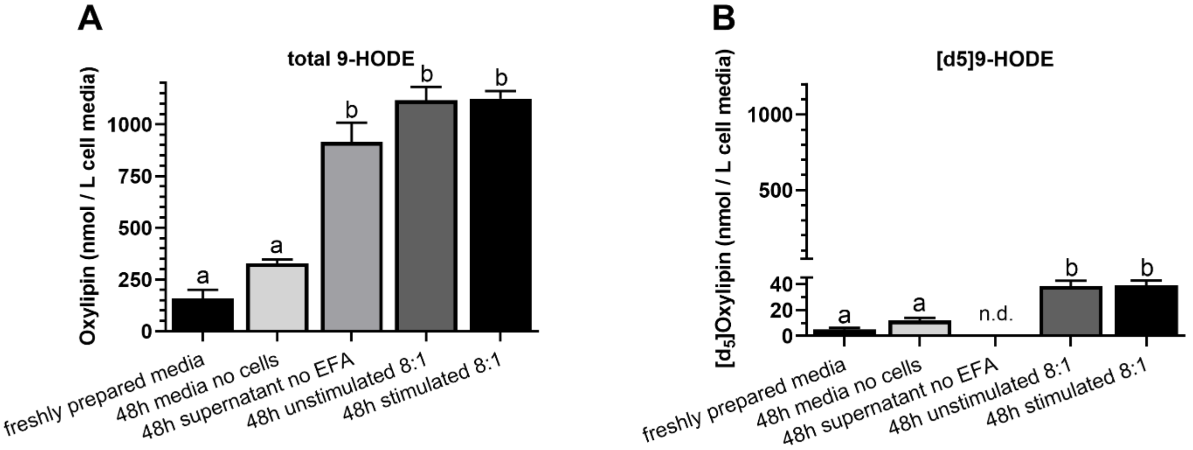

LC-MS/MS analysis of (A) unlabelled and (B) labelled 9-HODE in control culture media and supernatant of 48h cultured human CD3+ T cells with no EFA added or an EFA ratio of 8:1. Statistic analysis was performed in GraphPad Prism 8 with One-way unpaired ANOVA. Means with different letters were significantly different ($p < 0.05$). Samples were freshly prepared media and media maintained 37°C for 48h without T cells (n = 3 each), supernatant from approximately 1.5 x10^6 mitogen-stimulated T cells without addition of EFA to adjust the EFA ratio (n = 7); supernatant from ~1.5 x10^6 unstimulated or mitogen-stimulated T cells with adjusted fatty acid composition to produce an EFA ratio of 8:1 (n = 10 each).
